# Vicarious Rewards Modulate the Drift Rate of Evidence Accumulation From the Drift Diffusion Model

**DOI:** 10.3389/fnbeh.2019.00142

**Published:** 2019-07-02

**Authors:** Laure Bottemanne, Jean-Claude Dreher

**Affiliations:** Neuroeconomics, Reward and Decision-Making Team, Institut des Sciences Cognitives Marc Jeannerod, Centre National de la Recherche Scientifique, Bron, France

**Keywords:** social cognition, motivation, decision making, drift diffusion models, drift rate, vicarious reward

## Abstract

Taking other people’s interests into account is a fundamental ability allowing humans to maintain relationships. Yet, the mechanisms by which monetary incentives for close others influence perceptual decision-making processes remain elusive. Here, we compared perceptual decisions motivated by payoffs for oneself or a close relative. According to drift diffusion models (DDMs), perceptual decisions are made when sensory evidence accumulated over time – with a given drift rate – reaches one of the decision boundaries. We used these computational models to identify whether the drift rate of evidence accumulation or the decision boundary is affected by these two sources of motivation. Reaction times and sensitivity were modulated by three factors: the Difficulty (motion coherence of the moving dots), the Payoff associated with, and the Beneficiary of the decision. Reaction times (RTs) were faster for easy compared to difficult trials and faster for high payoffs as compared to low payoffs. More interestingly, RTs were also faster for self than for other-affecting decisions. Finally, using DDM, we found that these faster RTs were linked to a higher drift rate of the decision variable. This study offers a mechanistic understanding of how incentives for others and motion coherence influence decision-making processes.

## Introduction

When playing at a shooting range in a fairground, we accumulate sensory evidence (about target movement) until we can shoot accurately, and win the prize. Now, if such decisions are made so that the prize goes to a close friend, will we process and use information in the exact same way? More precisely, how does motivational incentives for someone else influence the mechanisms engaged in making simple perceptual choices as compared to the same decisions associated with the same incentives, but for you?

In the last decades, the framework of sequential-sampling models, such as drift diffusion models (DDMs), has proven to be a powerful approach to explain the process of making a decision (Vandekerckhove and Tuerlinckx, 2007; Ratcliff and McKoon, 2008; Leite and Ratcliff, 2010; Summerfield and Tsetsos, 2012; Forstmann et al., 2016; Ratcliff et al., 2016). DDMs successfully capture the complex relationship between choice and reaction times (RTs) by decomposing these behavioral data into internal cognitive components of decision processing. In this framework, a decision reflects a decision variable drifting with a given rate (*v*), from an intermediate starting point (*z*) toward one of the decision boundaries at hands. Each boundary is separated from the starting point (*z*) of a given distance (*a*) and acts as a decision threshold for an option, so that the response of a decision is initiated when the decision variable reaches one of the boundaries. In the example of the shooting range, the decision variable would accumulate information about the position of the moving ducks over time, and when (relative) certainty about their position is reached, the decision of pulling the target is made.

Sensory encoding of information basically relies on the quality of the available evidence (Ratcliff and McKoon, 2008). A foggy weather would slow the rate at which the decision variable rises, as compared to clear climate conditions. Reliability of the decision depends on the distance between the starting point of the decision variable and the decision boundary; the decision rules are set by the read-out mechanisms (Brainard, 1997; Summerfield and Tsetsos, 2012; Oppenheimer and Kelso, 2015; Forstmann et al., 2016). Reaching higher decision boundaries requires more evidence to be accumulated, thus leading to a better accuracy, but takes a longer time. Which of the evidence accumulation stage (drift of the decision variable) or the read-out mechanisms (distance between the starting point and the decision boundaries) would be adjusted differently based on vicarious information (the beneficiary of the decision)? How is the perceptual decision process modulated when the source of motivation concerns a close relative rather than oneself?

**FIGURE 1:**
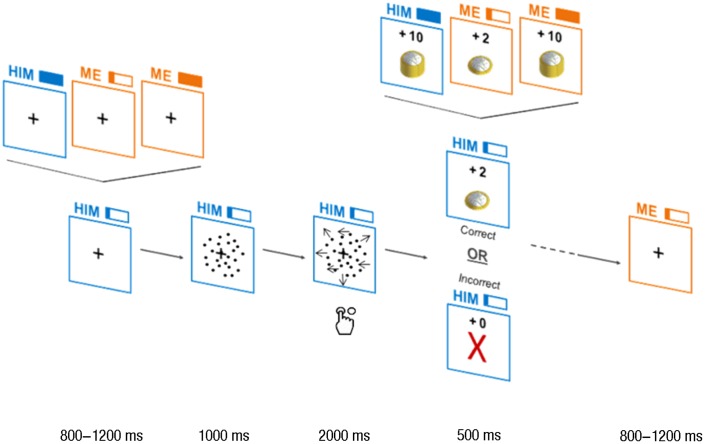
Trials design. Each trial began with a cue, showing “me” or “him” (for self- and other-affecting decisions, respectively) and a full filled rectangle (high payoff) or a one-fifth filled rectangle (low payoff) on top of a square. The cue and the square were depicted in yellow or in blue, according to the beneficiary. Then, the first frame of the random dots kinematogram (RDK; stationary dots) appeared in the square for 1,000 ms. Following this, the moving dots were presented for 2,000 ms and the subject had to respond while the dots were moving. At the end of the 2,000 ms of dots motion, the payoff was presented. If the response was correct, a pile of coins proportional to the payoff was shown together with the value of payoff itself (“+2,” “+10”) above it. For incorrect responses and misses, a red-colored cross was displayed together with “+0” on top of it. Then, a new trial began and the cue of the upcoming trial was shown.

**FIGURE 2:**
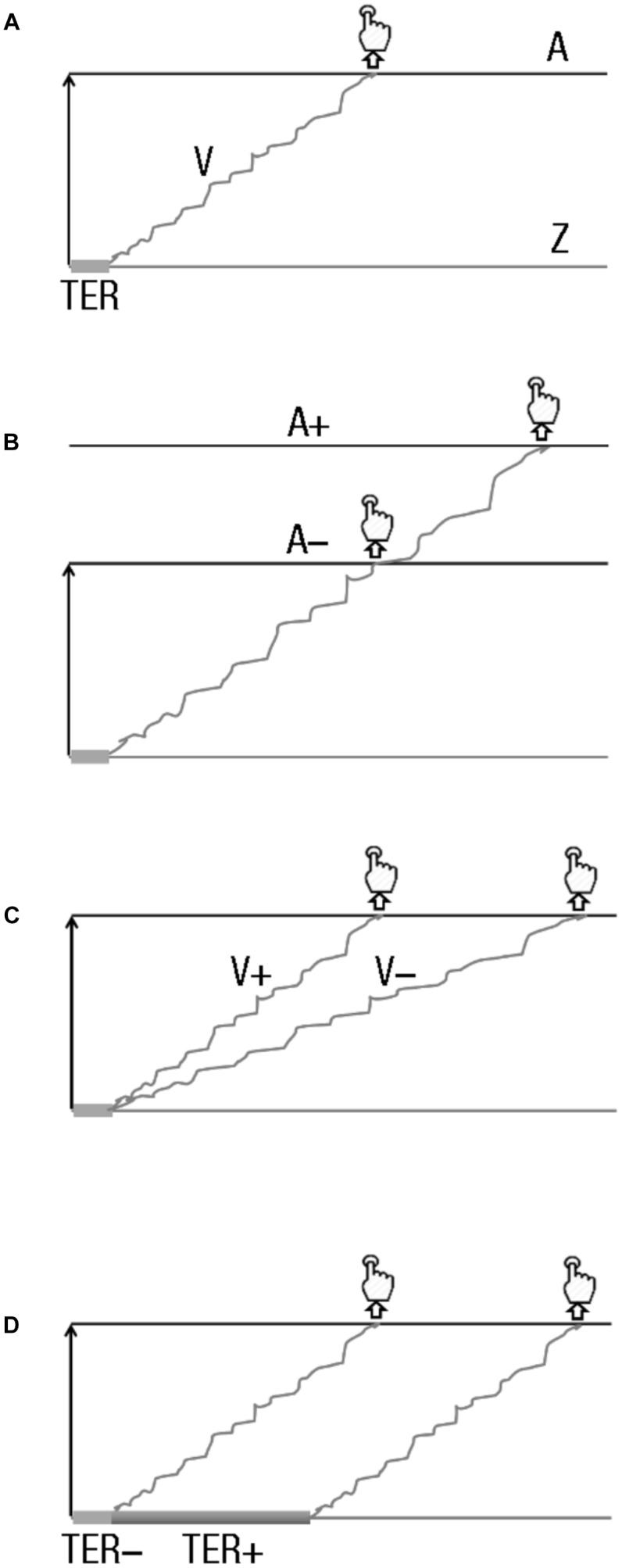
Drift diffusion models (DDMs) and hypotheses. **(A)** DDM main parameters. DDM assumes that two-choice decisions are made by a noisy process that accumulates information, with a given drift rate (*v*), over time. This process goes from the starting point (*z*) toward the decision boundary. When the boundary is reached, a response is initiated (with a button press, in usual experimental setups). The starting point and the boundary are separated by the distance (*A*). **(B–D)** Effects of boundary modulation, drift-rate changes, and non-decision time variation on response initiation. **(B)** Boundary modulation. A boundary increase (*A*+) leads decisions that require more sensory evidence, and thus a longer time, than when a lower boundary is set up (*A*–). **(C)** Drift-rate change. A drift rate increase (*V*+) produces faster sensory evidence accumulation than a lower one (*V*–), producing faster reaction times. **(D)** Non-decision time variation. A longer non-decision time (TER+) leads to slower decisions than a shorter one (TER–).

Here, we designed a new paradigm, enabling the use of DDMs to investigate the influence of the payoff associated with and the person affected by a perceptual decision (Figure 1). The participants performed a random dots task (left/right direction categorization) to win low or high payoffs, for themselves or for a close relative. We tested which of the DDM parameters are modified between other-affecting and self-affecting decisions: the drift rate of the decision variable (encoding) or the decision boundary (read-out; Figure 2)? Changes in the distance between the starting point and the decision boundary (*a*) would mean that people integrate beneficiary-related motivation through the read-out mechanisms, setting the decision rules prior to starting the evidence integration itself. Alternatively, a direct influence of self/other motivation on the decisional process could affect the drift rate of the decision variable, which is an index of the quality of evidence used for the decision. This would suggest that sources of motivation (payoff for self/payoff for other) are integrated together with the evidence for the choice alternatives into a single source of evidence during the accumulation process. Finally, a variation in the non-decision time would indicate that the beneficiary-related motivation acts on cognitive mechanisms outside of the decision process itself, such as primary encoding of the stimuli and motor execution.

## Materials and Methods

### Participants

Forty healthy subjects were recruited by advertisements in the Lyon 1 Claude Bernard University students’ mailing list. Subjects were screened using self-reports to exclude any psychiatric or neurological history, and current or previous substance abuse (except nicotine and festive alcohol consumptions). All participants gave written informed consent and received 20€ for their participation. This study was approved by the local research ethics committee (Comité de Protection des Personnes Sud-Est III); all methods were performed in accordance with the relevant guidelines and regulations. Two subjects were excluded, one for chance level performances and the other for technical problems, leaving 38 subjects for further analyses (15 females; mean age = 21.84, range = 18–34).

### Stimuli

Random dots kinematograms (RDKs) were programmed using the MATLAB^®^ Psychtoolbox (Brainard, 1997; Pelli, 1997). The mask stimulus was a drifting random dots display of 2000 ms duration. Dots were white on a black background, with each frame composed of 50 white Gaussian blobs with a diameter of 2.85 mm. The stationary dots began to move with a speed of 2.7°/s from their original locations, and each dot had a life duration of 500 ms. The motion of the dots was made by replotting dots corresponding to the previous ones at a determined spatial offset in the same direction so that all the dots moved in their directions at the same speed. During the experiment, RDKs appeared in a square centered on the screen (Dell, 19″, screen resolution set to 1,280 × 1,050, vertical refresh rate of 60 Hz), taking 30.8% of the screen, with participants at a distance of 60 cm.

### Procedure

Before going to the laboratory, the volunteers were asked to choose a close relative for who they would be willing to play for, on half of the experiment. At their arrival, the participants sat in the experimental room, were informed, and gave their written consent. Their relationship with the chosen person was asked [seven participants chose one of their parents (mother or father), seven chose a sibling, eight chose their lover, three chose a friend, and two chose their roommate]. A few demonstration trials were shown, for them to see how the condition cue (Payoff and Beneficiary) was displayed. Subjects were trained and then finally completed the task. It lasted approximately 64 min, in four blocks of 16 min each. All were debriefed when the task was over.

### Training

Before the task, subjects were trained to be familiarized with the design and timing. The training was composed of 10 trials of 15% coherently moving dots, which is the easy level of the task. To ensure that participants did not respond randomly, a sensitivity (*d*’) criterion was set at *d* = 0.6 (i.e., 60% correct, which is higher than chance level). If subjects were below this criterion in the training session, they performed a second identical training. All of the included subjects eventually reached the criteria and subsequently performed the task.

### Instructions

Participants were explained that they would perform a game in order to win money, either for themselves of for the close relative they chose. They were told that they would earn 10€ for doing the experiment and could win 2€ or 10€ more for themselves and also 2€ or 10€ for their relatives. The participants were asked to discriminate the left/right direction of coherently moving dots. They were instructed that they had to give one, and only one, response during the dots motion: if they gave more than one response or did not respond (miss), the program would consider it as incorrect. Money was not accumulated over trials, nor was such accumulation shown to the participants. They were told that one trial of each of the beneficiary condition (self and other) would be randomly selected (by a computer program) to determine their final payoffs. The payoff associated with the trial would be won by the beneficiary, if it was a correct trial. Participants were told (and believed) that the payoff for the other (as well as for themselves) would be sent after completing the experiment. In reality, the close relative received nothing and all participants received 20€ (as if the selected trial was won for himself and associated with a high payoff). This procedure (i) ensured that participants treated all decisions as equally relevant, both for themselves and their close relative; (ii) avoided any competition effects to arise between self and other interests. Also, accuracy was implicitly emphasized by telling the participants that, although they would have to adapt to the given 2 s to answer, time should not be a problem since the duration of the stimuli was chosen based on previous experimental results (pilot study).

### Task Design

A square was always present in the middle of the screen. On top of this square appeared the cue, which indicated the beneficiary and payoff conditions of the forthcoming trial. The dots were displayed inside the area defined by the square. The square and the cue were colored yellow or blue, according to the beneficiary of the payoff associated with the trial. The color was used to emphasize the beneficiary of the trial and was counterbalanced between subjects.

Each trial began with the cue, which had a jittered duration from 800 to 1,200 ms and was used as inter-trial interval (ITI). The cue consisted in a word announcing the beneficiary of the decision (“him” for others-affecting decisions, “me” for self-affecting decisions) to the left of a rectangle filled proportionally to the payoff associated with the decision (full filled rectangle for 10€, one-fifth filled rectangle for 2€). This cue remained on the screen during the entire subsequent trial. After the cue, the first frame of the RDK to come (a picture of stationary dots) was shown for 1,000 ms. Then, dots motion began and lasted for 2,000 ms, during which the subject had to respond. Motion coherence was either 13% (difficult) of 15% (easy), for all participants. At the end of the 2,000 ms of dots motion, the feedback illustrated the payoff for 500 ms. If the response was correct, a pile of coins proportional to the payoff (2 or 10€), was shown together with the value of payoff itself (“+2,” “+10”). For incorrect responses and misses, a red-colored cross was displayed together with “+0” above it. At the end of the trial, a new ITI was displayed, showing the cue for the trial to come.

A total of 104 trials per Beneficiary^∗^Payoff^∗^Difficulty condition were performed, leading to 832 trials per subject. The task was composed of 4 blocks, of 208 trials each. Each block included 26 trials of each of the 8 conditions. Difficulty levels, Payoffs, Beneficiaries, and dots direction were pseudo-randomized within each block and across participants. Randomization of dots direction was designed to avoid a bias toward one of the two (left or right) alternatives, constraining it to no more than three consecutive trials of the same dots direction.

It is to be noted that we actually ran a first experiment using another anonymous, randomly selected, participant as “the other.” However, there was no main effect of the beneficiary on RT or on *d*’ (Supplementary Table 1). Since we were aiming to characterize how others are taken into account into the perceptual decision-making process, and based on the literature showing that familiarity increase vicarious effects (Mobbs et al., 2009; Kawamichi et al., 2013), we adapted our task with a close relative.

### Statistical Analysis

Reaction times for corrects and RTs for errors were analyzed separately, and RTs were logarithmically transformed. logRT and sensitivity (*d*’) normality distribution was ensured using Lilliefors tests. logRT and *d*’ were then analyzed using three-way repeated-measures analyses of variances (rmANOVAs). The factors were as follows: “Beneficiary” (two levels: other vs. self), “Payoff” [two levels: high (10€) vs. low (2€)], and “Difficulty” [two levels: 13% motion coherence (difficult) vs. 15% coherence (easy)]. Beneficiary and Payoffs were overt factors, indicated by cues on each trial, but difficulty was not explicitly given to participants. During debriefing, we asked participants during debriefing how many difficulty levels they perceived. Most of them perceived two levels; only two of them thought there were more and one did not conscientiously perceived any. All *post hoc* analyses were performed using LSD Fisher tests. There was no effect of gender on behavior (Supplementary Table 2). Although there could be effects of sex hormone variations on decision making in young women, we did not record the phase of the menstrual cycle in our sample. All statistical analyses were performed using Statistica (STATISTICA^®^, Dell Inc., 2015), except for normality tests and DDM fitting, performed on MATLAB^®^.

### Fitting the DDM to the Data

The DDM assumes that two-choice decisions are made by a noisy process that accumulates information over time from a starting point (z) toward one of two choice criteria or boundaries (here, corresponding to left and right response decision, respectively; Figure 2A). When one of the boundaries is reached, a response is initiated. The starting point and the decision boundaries are separated by distance (*a*). The evidence that drives the accumulation process, the drift rate (*v*), is derived from the representation of the stimulus. The better the quality of the evidence, the larger the drift rate toward the appropriate decision boundary, and the faster and more accurate the response (Figure 2C). The components of processing acting outside the decision process itself, such as encoding and response output, are combined in a single parameter: the non-decision time parameter (Ter). RT being the result of non-decision time added to the time it takes for accumulated evidence to reach one of the boundaries, and sensitivity coming from the reached boundary that determines which response is given, the model extracts the components of the decision process (values of drift rate, non-decision processes, and boundaries) from RT distribution and sensitivity data simultaneously.

For fitting the diffusion model to the data (Ratcliff and Tuerlinckx, 2002; Vandekerckhove and Tuerlinckx, 2007), we used the MATLAB Diffusion Model Analysis Toolbox [DMAT (Vandekerckhove and Tuerlinckx, 2008)]. The DMAT extracts the components of the decision process and their variability from RT distribution and sensitivity data from all trials for each condition. All trials, correct and error, are thus included in the DMAT parameter estimation. Parameters are estimated by maximizing a multinomial likelihood function. Left and right trials being equally distributed across the experiment (50% of trials for each direction, within each block), the underlying diffusion processes are supposed to be symmetric and no bias toward the left or right answer should arise. We ran a model where the starting point (*z*) was estimated independently from the decision boundary for the left and the right button presses separately. We then checked that *z* was not different between left and right responses using a one-way rmANOVA with response direction as factor. The analyses showed no effect of response direction (*F*_1,37_ = 0.001; *p* = 0.971), ensuring that no bias emerged toward either the left or the right response. Consequently, we applied in all our models a starting point equal to half the distance between the left and right decision criteria (*z* = ½ *a*). Each model was fitted to the data separately for each participant.

The first model we ran allowed all three parameters to vary [the boundary (*a*), the drift (*v*), and the non-decision time (Ter)]. The estimated parameter values did not follow a normal distribution; we thus used a decimal logarithmic transformation and ensured it normalized their distribution using Lilliefors tests (Supplementary Table 3) before we applied the three-way rmANOVA. The three factors were the Beneficiary of the decision, the Payoff associated with the decision and the Difficulty (dots coherence). The boundary (*a*) and the non-decision time (Ter) showed no effect of any factor. We thus ran a model where only the drift (*v*) was free to vary across conditions. Once again, we analyzed log(*v*) using the same three-way rmANOVA. In order to compare the goodness of fit of our models, we also ran the intermediate models (either the drift and the boundary or the drift and the non-decision time were allowed to vary) and compared the sum of the individual Bayesian Information Criterion (BIC) of the models.

### Data Availability

The data used in the present paper will be available to any reader after publication. The datasets generated and/or analyzed during the current study will be available in the repository, on a permanent free-access web link.

## Results

Participants performed a random dots (left/right direction categorization) task to win low or high payoffs, for themselves or for a close relative. RTs and sensitivity (*d*’) were collected and analyzed using three-way rmANOVAs, with “Beneficiary” (two levels: Other vs. Self), “Payoff” (two levels: High vs. Low), and “Difficulty” (two levels: Difficult vs. Easy) as factors.

**FIGURE 3:**
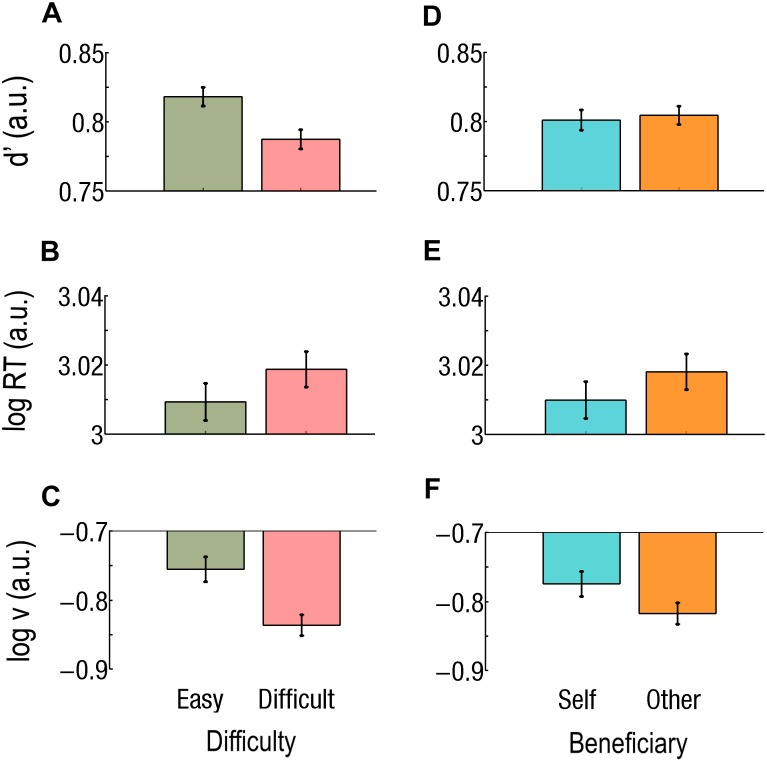
**(A–C)** Main effect of difficulty. Easy trials are green and on the left, Difficult trials are red and on the right. **(A)** Sensitivity (*d*’) is worse, **(B)** reaction times (RTs) are longer, and **(C)** drift rate (*v*) is lower during Difficult trials than during Easy trials. **(D–F)** Main effects of beneficiary. Self-affecting trials are cyan and on the left; other-affecting trials are orange and on the right. **(D)** Sensitivity did not differ but there was **(E)** a faster RT and **(F)** a higher drift rate (*v*) for Self than for Others. Log RT, *d*’, and log *v* are expressed in arbitrary units (a.u.). Bars represent the standard errors of the mean (SEM).

### Sensitivity (*d*’)

Participants missed only one trial in the experiment. A main effect of task Difficulty was found; *d*’ was better during Easy trials than during Difficult trials (*d*’_Easy_ = 0.82; *d*’_Difficult_ = 0.79; *F*_1,37_ = 57.4; *p* = 0.0000001; Cohen’s *d* = 0.362; Figure 3A). All interaction effects also reached significance, including the triple interaction effect (*F*_1,37_ = 16.8; *p* = 0.000220). We consequently ran two-ways rmANOVAs for each difficulty level, keeping Beneficiary and Payoff as factors.

**FIGURE 4:**
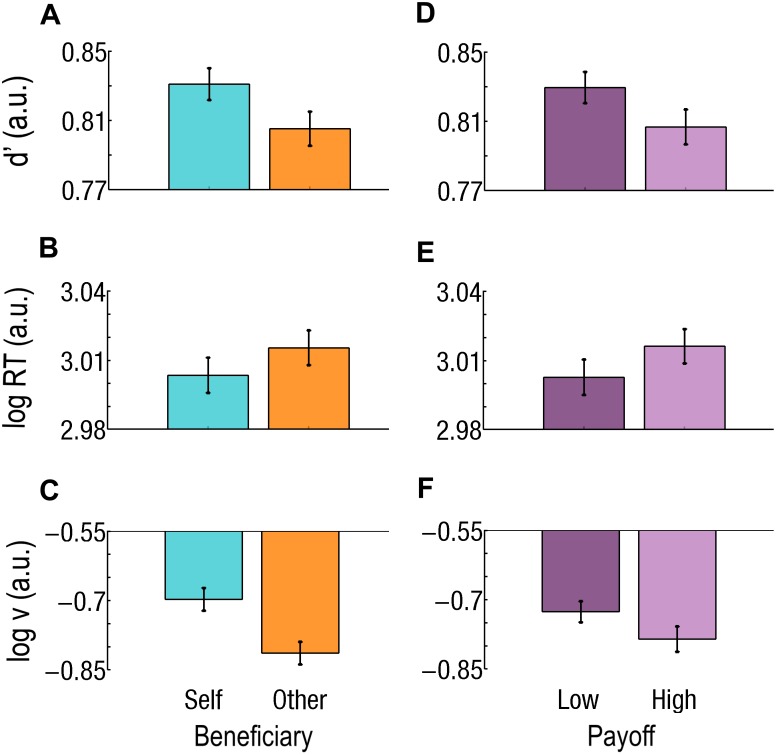
Effect of beneficiary and payoff for easy trials only. **(A–C)** Beneficiary. Self-affecting trials are cyan and on the left; Other-affecting trials are orange and on the right. During Easy trials, **(A)** sensitivity (*d*’) is better, **(B)** RTs are faster, and (C) drift rate (*v*) is higher for Self than for Other. **(D–F)** Effect of payoff. Low Payoffs are in deep purple and on the left; High Payoffs are in light purple and on the right. **(D)** Sensitivity (*d*’) is better, **(E)** RT is faster, and **(F)** *v* is higher for Low than for High Payoffs. Log RT, *d*’, and log *v* are expressed in arbitrary units (a.u.). Bars represent the standard errors of the mean (SEM).

**FIGURE 5:**
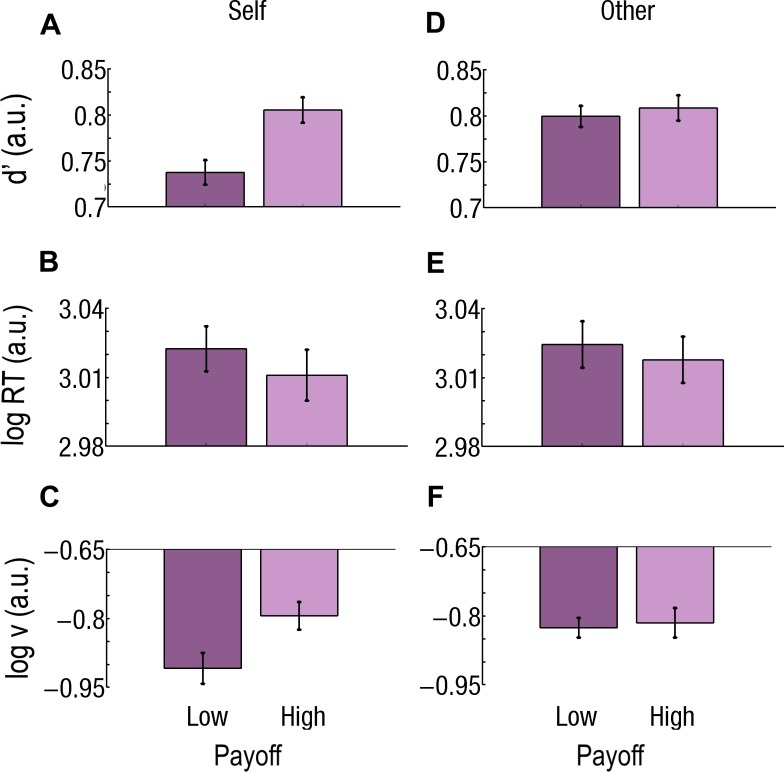
Effect of payoff for difficult trials for self and close relative (other). **(A–C)** Effect of payoff for Self. **(A)** Sensitivity (*d*’) is lower, **(B)** reaction times (RTs) are slower, and **(C)** drift rate (*v*) is lower for Low Payoffs than for High Payoffs. **(D–F)** Effect of payoff for Other. There is no difference in **(D)** sensitivity (*d*’), **(E)** RT, or **(F)** *v* between Low and High Payoffs. Log RT, *d*’, and log *v* are expressed in arbitrary units (a.u.). Bars represent the standard errors of the mean (SEM).

During Easy trials, *d*’ was better for Self than for Other (*d*’_Self_ = 0.83; *d*’_Other_ = 0.80; *F*_1,37_ = 16.2; *p* = 0.000276; Cohen’s *d* = 0.305; Figure 4A) and better for Low than for High Payoffs (*d*’_Low_ = 0.83; *d*’_*High*_ = 0.81; *F*_1,37_ = 11.5; *p* = 0.001683; Cohen’s *d* = 0.266; Figure 4D). During Difficult trials, both Beneficiary (*d*’_Self_ = 0.77; *d*’_Other_ = 0.80; *F*_1,37_ = 24.7; *p* = 0.000015; Cohen’s *d* = 0.375) and Payoff (*d*’_Low_ = 0.77; *d*’_*High*_ = 0.81; *F*_1,37_ = 30.0; *p* = 0.000003; Cohen’s *d* = 0.465) were significant. The Beneficiary^∗^Payoff interaction also reached significance (*F*_1,37_ = 19.9; *p* = 0.000072). Sensitivity for Self-affecting decisions associated with a Low Payoff was lower than for Other-affecting ones (*d*’_Self_ = 0.74; *d*’_Other_ = 0.80; *p* < 0.000001; Cohen’s *d* = 0.780) and lower than when associated with a High Payoff (Self: *d*’_*High*_ = 0.80; Other: *d*’_*High*_ = 0.81; *p* < 0.000001; Cohen’s *d* = 0.816; Figure 5A).

### Reaction Times

The results presented here come from analyses performed on logarithmically transformed RTs (decimal logarithm), for correct and error trials separately. For intelligibility, the mean values in the following paragraph are given as non-transformed RT, in milliseconds (ms). Difficulty had an effect on log RT from errors, with subjects being slower during Difficult than during Easy trials (RT_Difficult_ = 1146 ms; RT_Easy_ = 1110 ms; *F*_1,37_ = 6.6; *p* = 0.0146; Cohen’s *d* = 0.209). This was the only effect on RT from errors.

All the following results concern correct responses. We found a main effect of task Difficulty (Figure 3B) and a main effect of Beneficiary (Figure 3E) on log RT (for correct responses). That is, RTs were slower during Difficult than during Easy trials (RT_Difficult_ = 1055 ms; RT_Easy_ = 1033 ms; *F*_1,37_ = 36.56; *p* < 0.001; Cohen’s *d* = 0.144) and slower for Other than for Self (RT_Other_ = 1054 ms; RT_Self_ = 1035 ms; *F*_1,37_ = 18.86; *p* < 0.001; Cohen’s *d* = 0.125). The triple interaction effect was not significant (*F*_1,37_ = 0.22; *p* = 0.645). However, both the Beneficiary^∗^Difficulty and the Payoff^∗^Difficulty interaction effects reached significance (*F*_1,37_ = 37.10; *p* < 0.000001 and *F*_1,37_ = 4.26; *p* = 0.0461, respectively). Given the main effect of Difficulty, we then ran separate two-way rmANOVA at each Difficulty levels, keeping Beneficiary and Payoff as factors.

RTs were slower for Other than for Self, during Easy trials only (RT_Other_ = 1047 ms, RT_Self_ = 1020 ms, *F*_1,37_ = 32.6; *p* = 0.000002; Cohen’s *d* = 0.180; Figure 4B). Payoff had an effect at both Difficulty level, but with opposite direction. During Easy trials, RTs were slower for High than for Low Payoffs (RT_*High*_ = 1049 ms, RT_Low_ = 1017 ms, *F*_1,37_ = 23.5; *p* = 0.000022; Cohen’s *d* = 0.203; Figure 4E), while during Difficult trials, they were faster for High than for Low Payoffs (RT_*High*_ = 1045 ms, RT_Low_ = 1065 ms, *F*_1,37_ = 21.53; *p* = 0.000043; Cohen’s *d* = 0.142).

### DDM Parameters

We started with the selection of the best-fitting model. The first model we ran allowed all three parameters [the boundary (*a*), the drift (*v*), and the non-decision time (Ter)] to vary. In this model (“full model”), the boundary (*a*) and the non-decision time (Ter) showed no effect of any of the three factors (Beneficiary, Payoff, and Difficulty). We thus applied a model where only the drift (*v*) was free to vary across conditions (“*v* free”). In order to compare the goodness of fit of our models, we also ran the intermediate models (either the drift and the boundary, “*v* free–*a* free,” or the drift and the non-decision time, “*v* free–Ter free,” were allowed to vary) and compared the sums of the individual BIC of the models. The model where only the drift (*v*) was allowed to vary showed a lower BIC than all other models (BIC sums: full model: 7.62 × 10^4^, *v* free: 7.30 × 10^4^; *v* free–*a* free: 7.46 × 10^4^; *v* free–Ter free: 7.45 × 10^4^). To ensure that this reflected individual fits, we also compared the BICs of the models within each individual. Thirty-six of 38 subjects were best fitted with the model where only the drift is allowed to vary (“*v* free”); the two other subjects were best fitted with the addition of modulations of the boundary *a* (“*v* free–*a* free”). Furthermore, we ran the simulations of the data predicted by the model using the estimated parameter, for each subject (Supplementary Figure 1).

We subsequently applied a three-way (Beneficiary, Payoff, and Difficulty) rmANOVAs on the drift parameter (*v*) from the “*v* free” model. Note that log(*v*) values are negative, so that higher absolute values of log(*v*) actually mean lower drift rates (*v*) of the decision variables. Difficulty had a main effect on the drift rate (*v*), which was higher during Easy than during Difficult trials [log(*v*)_Easy_ = −0.76; log(*v*)_Difficult_ = −0.84; *F*_1,37_ = 35.9; *p* = 0.000001; Cohen’s *d* = 0.503; Figure 3D]. Beneficiary also had a main effect, *v* being higher during Self- than during Other-affecting decisions [log(*v*)_Self_ = −0.78; log(*v*)_Other_ = −0.82; *F* = 4.42; *p* = 0.0423; Cohen’s *d* = 0.273; Figure 3F]. The Beneficiary^∗^Payoff interaction also reached significance (*F*_1,37_ = 6.28; *p* = 0.01673). For decision associated with a High Payoff, *v* was higher for Self than for Other [log(*v*)_Self_ = −0.76; log(*v*)_Other_ = −0.83; *p* = 0.000078; Cohen’s *d* = 0.385]. The Beneficiary^∗^Difficulty and the Payoff^∗^Difficulty interactions were significant (*F*_1,37_ = 29.5; *p* = 0.0000004 and *F*_1,37_ = 13.3; *p* = 0.000801, respectively). We consequently ran two-way rmANOVAs at each Difficulty level, keeping Beneficiary and Payoff as factors.

During Difficult trials, Payoff had a main effect [log(*v*)_*High*_ = −0.81, log(*v*)_Low_ = −0.87, *F*_1,37_ = 9.28; *p* = 0.004265; Cohen’s *d* = 0.409; Figure 5C], but the Beneficiary^∗^Payoff interaction was also significant (*F*_1,37_ = 8.80; *p* = 0.005251). Payoff actually had an effect only for Self-affecting decisions, with a higher drift (*v*) for High than for Low Payoffs [log(*v*)_*High*_ = −0.80, log(*v*)_Low_ = −0.91; *p* = 0.000045; Cohen’s *d* = 0.592].

During Easy trials, both Beneficiary and Payoff had a main effect: the drift (*v*) was higher for Self than for Other [log(*v*)_Self_ = −0.70; log(*v*)_Other_ = −0.81; *F*_1,37_ = 19.8; *p* = 0.000076; Cohen’s *d* = 0.587] and higher for Low than for High Payoffs [log(*v*)_*High*_ = −0.79, log(*v*)_Low_ = −0.73, *F* = 6.18; *p* = 0.017588; Cohen’s *d* = 0.179].

## Discussion

Taking advantage of the DDM and the perceptual decision-making framework, we provided a mechanistic explanation of how others are integrated into the decisional process. Our results indicate that the beneficiary of the incentive associated with a decision modifies how decisions are performed. Decisions were faster for self than for others. As explained by the DDM, this was related to a higher drift rate (*v*) of the decision variable. In the present experiment, better sensitivity and faster RT were mirrored by higher drift rates. Higher drift rates have been found to explain shorter RT in tactile discrimination as well (Mulder and van Maanen, 2013). A change in the drift rate of the decision variable indicates a modification of the integration process itself, as branding does for economic value-based choices (Philiastides and Ratcliff, 2013). Our result indicates that sensory evidence is integrated faster for self than for others. In the example of the shooting range, if we aim to reach a target to win a price for a close relative, the decision process would not differ in the amount of evidence we would accumulate before making the decision to shoot, but rather in the efficiency of accumulation of the sensory evidence.

It may be that participants tried to imagine their relative receiving the payoff, although not instructed to do so. This would have required higher cognitive demands and redirect part of the attentional load and neuronal energy from the evidence accumulation process. Using the Game Theory and Public Good Games, studies show that taking into account another person into a decision engages the processes of *mentalizing* (or the Theory of Minds) (Frith and Singer, 2008; Stallen and Sanfey, 2013). It could also be that, when performing a self-affecting decision, more attentional resources are spent on the task (because of a higher motivation, due to direct self-benefit), thereby increasing the efficiency of evidence accumulation. In a study on value-based decision making combined with DDM, it has been suggested that, when choosing on behalf of another, a dual process takes place. Stimulus value integration, reflected in the drift rate (*v*), would be firstly computed based on self-preferences and then adjusted to the other’s inferred preferences (Harris et al., 2018). For others with similar preferences, RTs were longer and linked to a change in drift rate. Analogous mechanisms could have occurred during our experiment as well. The importance accorded to the evidence, reflected in the drift rate (*v*) of the decision variable, could have been initially lower during other-affecting decisions, or it could have been re-adjusted during the time of the decision. Alternatively, RTs for dissimilar others were also longer but associated with a higher decision boundary (*a*), which could have been implemented to overcompensate for an increased uncertainty about their preferences (Harris et al., 2018).

Payoffs for others could have been integrated into the perceptual decision process through a change in the decision rules, outside of the mechanism of sensory evidence accumulation and change the distance between the starting point of the decision variable and the decision boundary. Other researchers also suggested that payoff can modify both stages, evidence accumulation and decision boundary. It postulates two processes, one for payoffs and another for stimulus information, and that on a given trial, attention is directed toward one of these information, never both (Diederich and Busemeyer, 2006; Diederich, 2008). Sequential-sampling models have previously been used to account for the effects of payoffs in a perceptual decision task with time constraints. These studies have reported changes in the distance from the starting point to the decision boundaries, a bias in the starting point of the decision variable, induced either by prior probabilities of being correct (Leite and Ratcliff, 2010; Mulder et al., 2012) or by asymmetrical payoffs associated with the possible response alternatives (Simen et al., 2009; Mulder et al., 2012). These changes were characterized by a shift of the starting point of the decision variable closer to the decision boundary associated with the alternative having the higher probability or associated with the higher payoff. The starting point is then further from the other boundary (for the other alternative at hand) and the decision variable is less likely to reach it, establishing a bias and a change in response proportion.

In contrast, our experimental setup was designed to avoid response probability manipulations toward one of the (left or right) alternatives, in terms of probability (through trials randomization) and in terms of payoff (by assigning the same payoff to both response alternatives). We aimed to compare identical decisions made by the participants, either for themselves or for another person. It would be interesting to adapt our paradigm to asymmetrical alternatives, with the payoff going to one of the beneficiaries depending on the correct answer. Following our results, it could be expected that a bias toward the response associated with self-payoff would emerge. Finally, a variation in the non-decision time (Ter) would have indicated that the beneficiary-related motivation acts on cognitive mechanisms that are outside of the decision process itself, such as primary encoding of the stimuli and motor execution. Non-decision time is usually referred to as reflecting the early encoding of the stimulus of interest and the execution of the motor response, once the decision process is completed (Brainard, 1997; Frith and Singer, 2008; Ratcliff and McKoon, 2008; Philiastides and Ratcliff, 2013; Stallen and Sanfey, 2013), both external to the visuo-motor decision process in itself. Moreover, the non-decision time is thought to be necessary to account for speed–accuracy trade-offs (Mulder and van Maanen, 2013), and it has been shown that speed–accuracy instructions also modulate the non-decision time (Zhang and Rowe, 2014). Variation in the non-decision time can mean that different strategies are applied (Schuch, 2016) and could include other components that influence the decision-making processes. However, the DDM cannot distinguish between different mechanisms within the non-decision time.

This study is a first step toward a better comprehension of how others influence decision-making processes. Altogether, our results suggest that the beneficiary affected by the decision is integrated together with the sensory evidence into the decision variable and affect the efficiency of the accumulation process during perceptual decision making. The present work provides further evidence of the strength of sequential-sampling models in a unified theory of choices (Summerfield and Tsetsos, 2012; Polanía et al., 2014, 2015), with outcomes that are self-interested or vicarious. However, while the main effect of beneficiary was significant on RT and drift rate (*v*), when analyzing difficulty levels separately, the effect was not present during difficult trials. This may be attributed to the fact that sensory evidence was too low for the drift to be modulated. Although the study of payoff *per se* was not our main goal, it is puzzling to observe that its effect was reversed between the easy and difficult level. Further studies are needed to confirm both results. A future direction would also be to specify how social distance to others changes perceptual decisions, as previously investigated using economic games where participants chose between selfish and generous alternatives (Strombach et al., 2015).

## Ethics Statement

All participants gave written informed consent and received 20€ for their participation. This study was approved by the local research ethics committee (Comité de Protection des Personnes Sud-Est III), all methods were performed in accordance with the relevant guidelines and regulations.

## Author Contributions

J-CD and LB designed the study and wrote the manuscript. LB collected the data and conducted the data analysis.

## Conflict of Interest Statement

The authors declare that the research was conducted in the absence of any commercial or financial relationships that could be construed as a potential conflict of interest.
