# Supplementary material for: Vicarious Rewards Modulate the Drift Rate of Evidence Accumulation From the Drift Diffusion Model
Source: Front Behav Neurosci. 2019 Jul 2;13:142. doi: 10.3389/fnbeh.2019.00142 (PMC6614513; doi:10.3389/fnbeh.2019.00142)
Supplement: Supplementary file 1 [file Data_Sheet_1.docx]

Supplementary Material

**Supplementary Figure 1.**


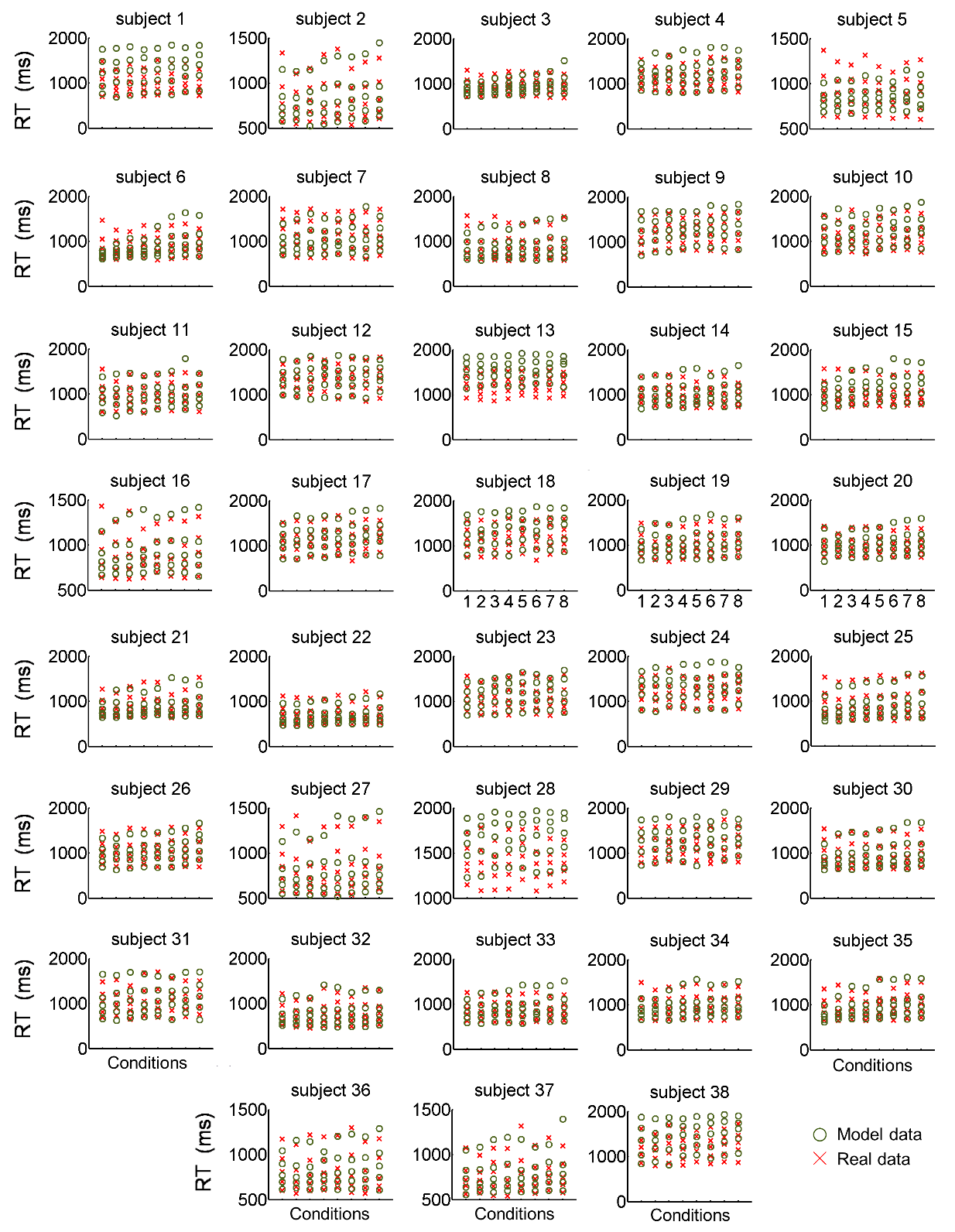


**Single subject quantile probability plots.** Quantile reaction times (RTs) for the .1, .3, .5, .7, and .9 quantiles (stacked vertically) are plotted for each experimental condition and for each subject. Predicted RTs (model data) are represented by the green circles, and collected RTs (real data) are the red crosses. RTs are expressed in milliseconds (ms).

**Supplementary Table 1.**

| **Statistics**  **Factor** |  |  | |  |  |  |
| --- | --- | --- | --- | --- | --- | --- |
|  | **logRTc** | | | | **d’** | |
| **BEN** | F_1,32 =_ 2.09 | | p = .158 | | F_1,32 =_ 1.69 | p = .203 |
| **PAY** | F_1,32 =_ 0.67 | | p = .418 | | F_1,32 =_ 2.61 | p = .116 |
| **DIF** | F_2,64 =_ 34.7 | | p < .000001 | | F_2,64 =_ 25.1 | p < .000001 |
| **BEN * PAY** | F_2,64 =_ 0.66 | | p = .423 | | F_2,64 =_ 0.09 | p = .766 |
| **BEN * DIF** | F_2,64 =_ 0.03 | | p = .971 | | F_2,64 =_ 0.95 | p = .392 |
| **PAY * DIF** | F_2,64 =_ 7.26 | | p = .0014 | | F_2,64 =_ 4.54 | p = .014 |
| **BEN * PAY * DIF** | F_2,64 =_ 4.01 | | p = .023 | | F_2,64 =_ 1.99 | p = .144 |

**When the other is anonymous, the main effect of beneficiary is not significant on reaction times and on sensitivity** (*d*’). There were three difficulty levels (13%, 15%, and 17% of dot coherence) in this version. Otherwise, factors were identical. The analysis was performed on logarithmically transformed reaction times from correct trials (logRTc) on 33 participants. BEN: Beneficiary; PAY: Payoff; DIF: Difficulty.

**Supplementary Table 2.**

| **Statistics**  **Factor** |  |  |  |  |
| --- | --- | --- | --- | --- |
|  | **logRTc** | | **d’** | |
|  | **F_1,37_** | **p** | **F_1,37_** | **p** |
| **Gender** | 1.10 | .302 | .075 | .785 |
| **BEN * Gender** | 2.26 | .141 | .378 | .543 |
| **PAY * Gender** | 1.23 | .274 | .235 | .631 |
| **DIF * Gender** | 1.14 | .294 | .253 | .618 |
| **BEN * PAY * Gender** | 0.00 | .992 | .636 | .430 |
| **BEN * DIF * Gender** | 0.16 | .689 | .515 | .478 |
| **PAY * DIF * Gender** | 1.24 | .273 | 1.35 | .253 |
| **BEN * PAY * DIF * Gender** | 1.75 | .194 | .261 | .612 |

**There was no effect of gender on reaction times (RT) or on sensitivity (*d*’)**, and in interaction with any of our three factors (BEN: Beneficiary; PAY: Payoff; DIF: Difficulty). The analysis was performed on logarithmically transformed reaction times from correct trials (logRTc).

**Supplementary Table 3.**

|  | ***Condition*** | | | | | | | |
| --- | --- | --- | --- | --- | --- | --- | --- | --- |
|  | **Self** | | | | **Other** | | | |
|  | **Low** | | **High** | | **Low** | | **High** | |
|  | **Easy** | **Difficult** | **Easy** | **Difficult** | **Easy** | **Difficult** | **Easy** | **Difficult** |
| **v** | .049 | .061 | .233 | > .5 | .232 | .001 | .001 | .017 |
| **a** | .284 | > .5 | .035 | .405 | .368 | .001 | > .5 | .005 |
| **Ter** | .003 | .193 | .007 | .084 | .289 | .004 | .088 | .003 |
| **Log(v)** | .273 | > .5 | > .5 | .301 | > .5 | .210 | .459 | .004 |
| **Log(a)** | > .5 | .298 | > .5 | .195 | .063 | .485 | .052 | .151 |
| **Log(Ter)** | .140 | > .5 | > .5 | .001 | > .5 | .389 | > .5 | .194 |

**Log transformation effectively normalized parameter distributions.** *P* values of the (Lilliefors) normality tests performed on the DDM estimated parameters and their logarithmic transformation. *v*: drift rate; *a*: boundary; Ter: non-decision time. Log(): logarithmic transformation.
